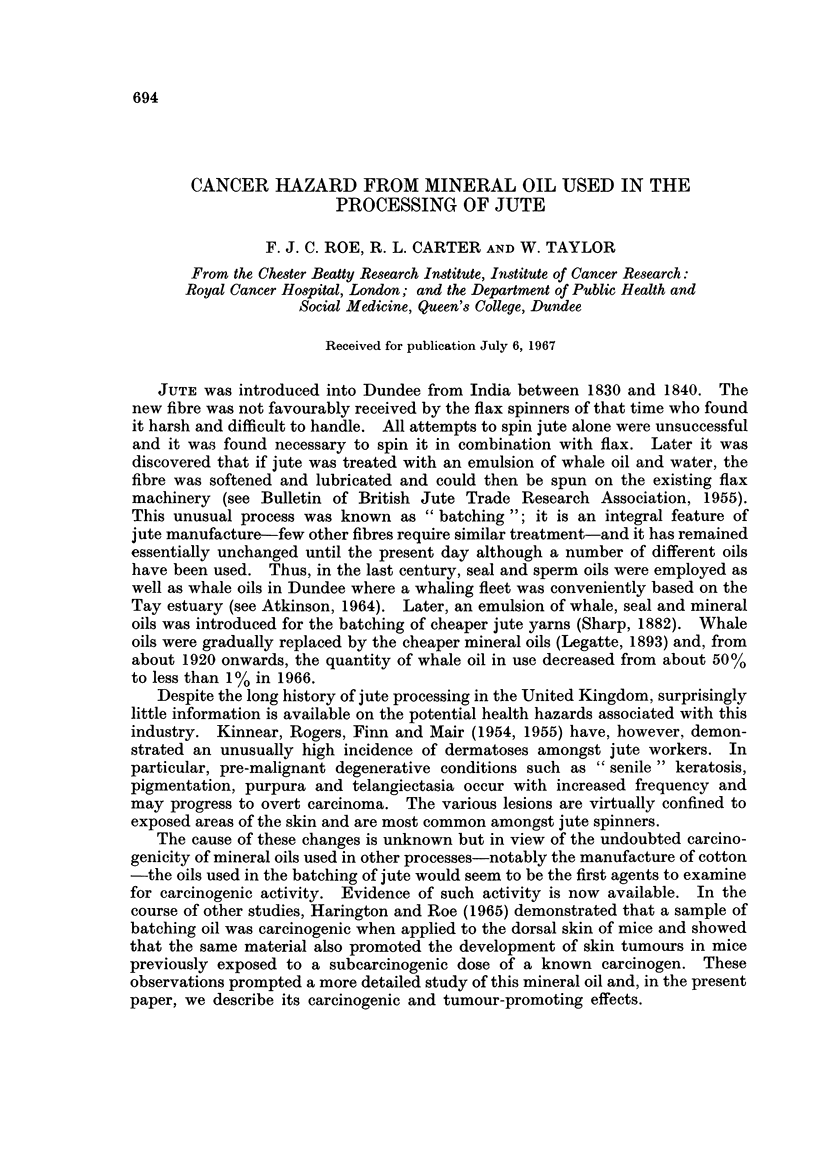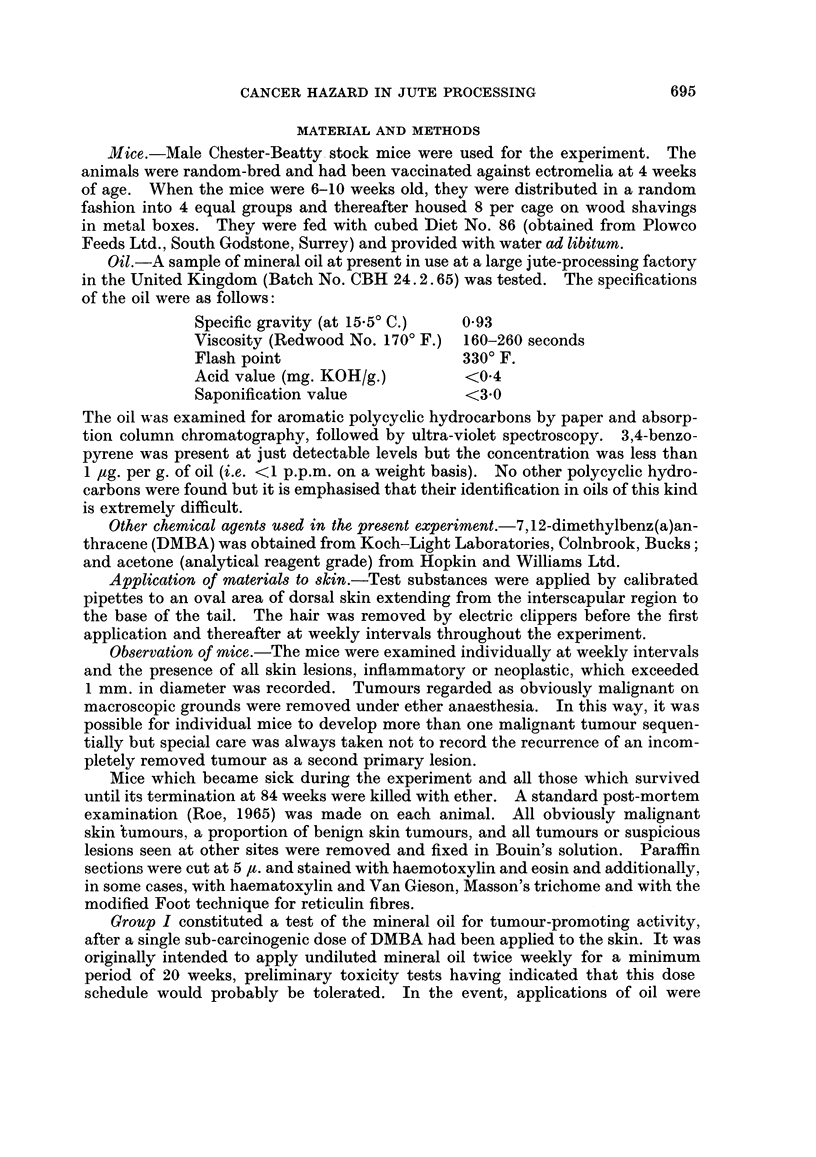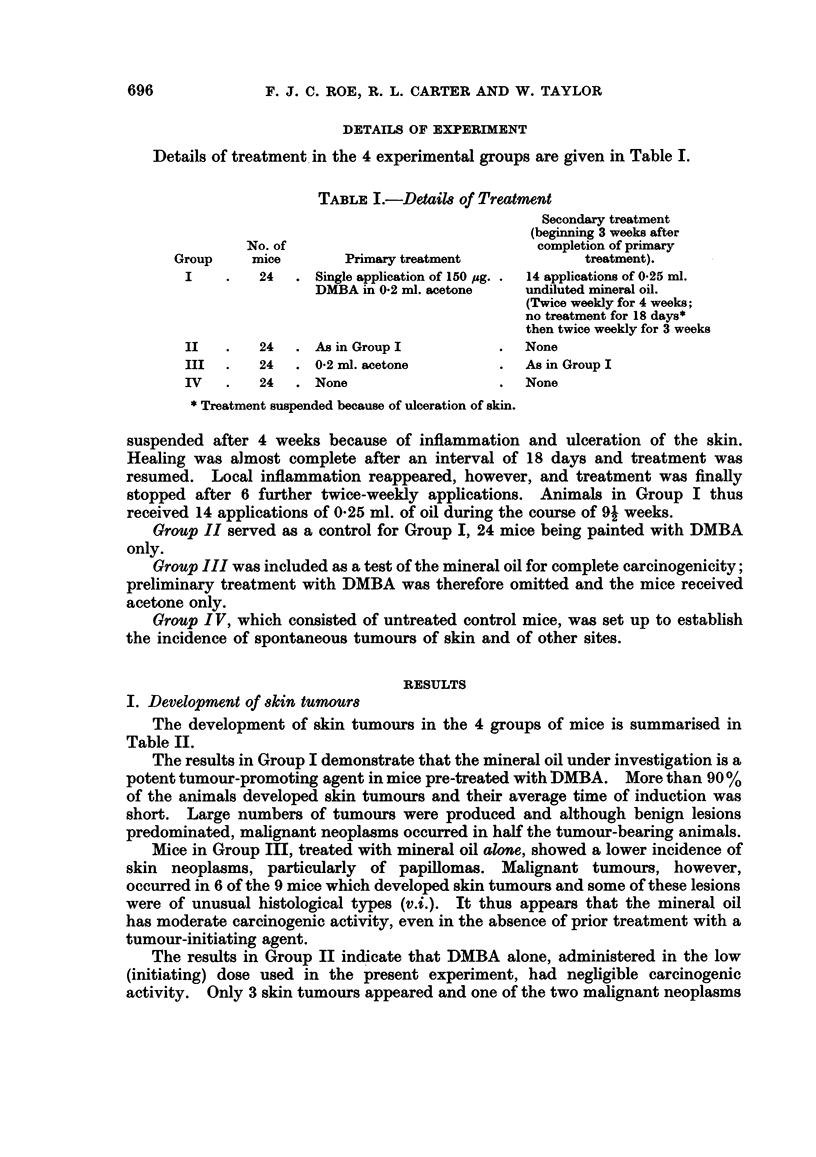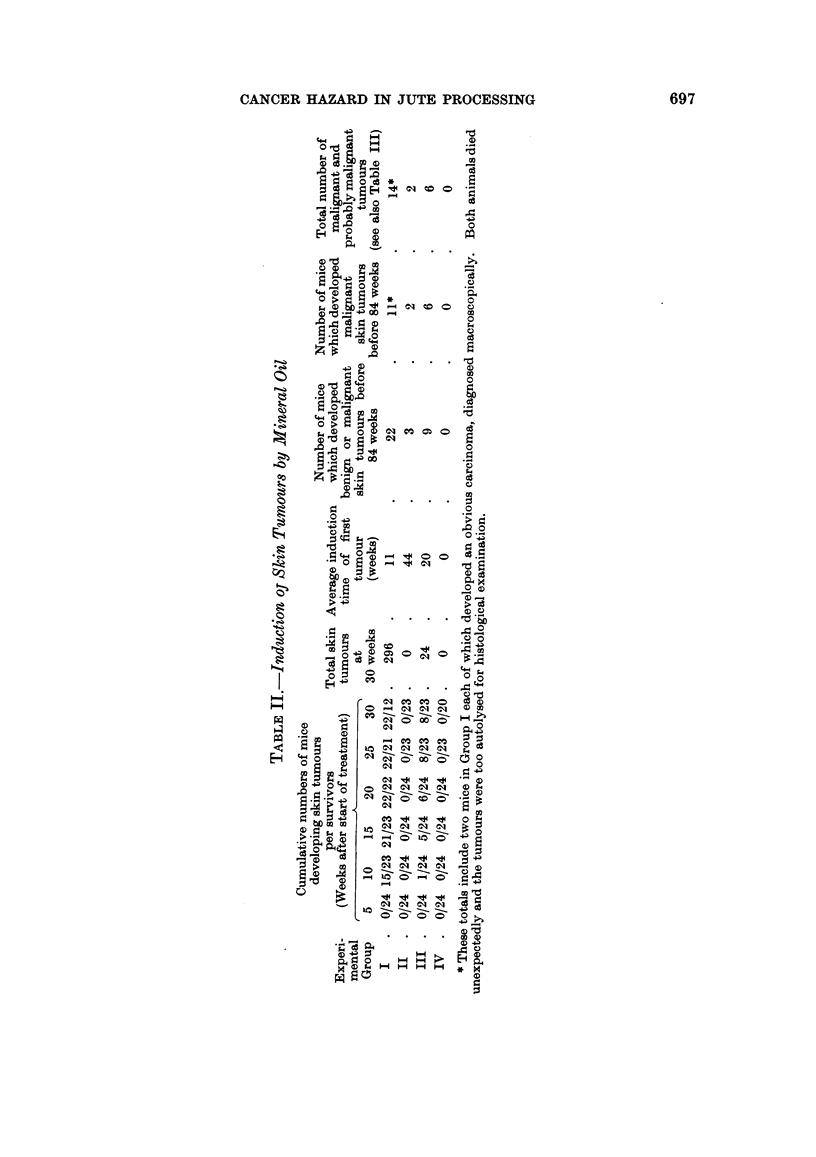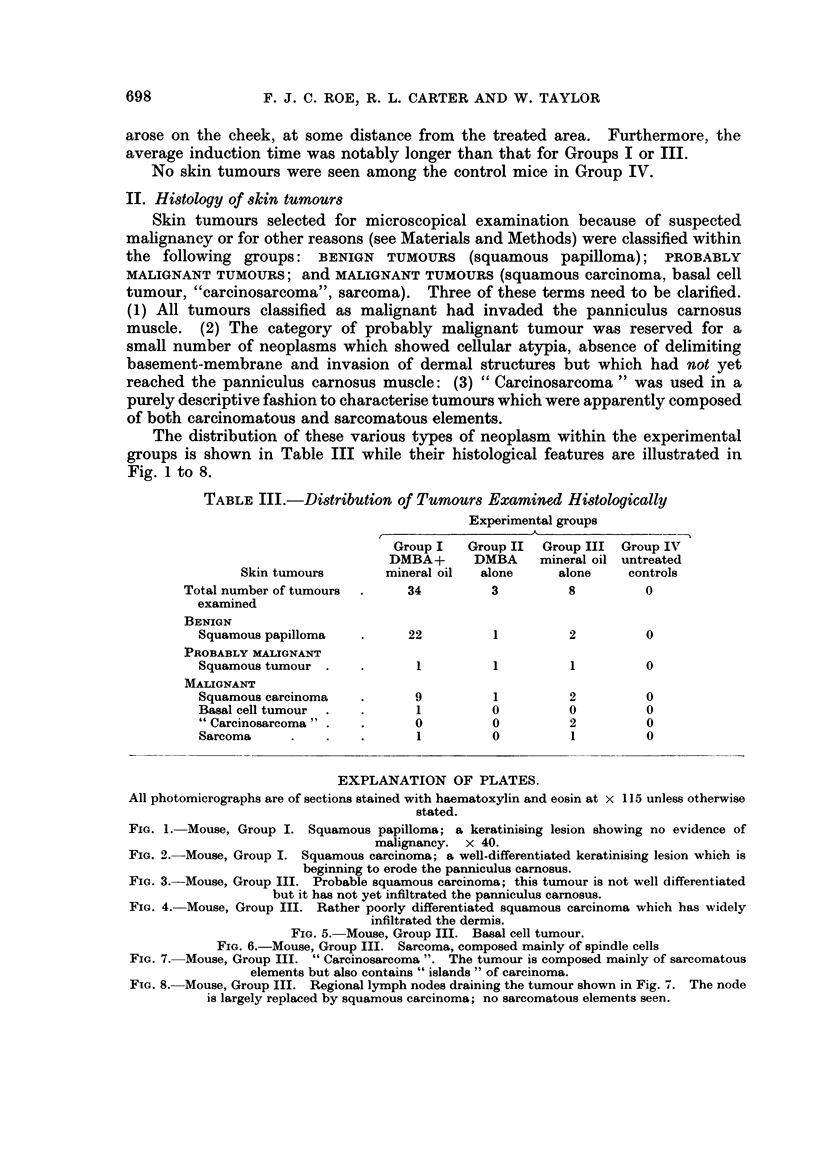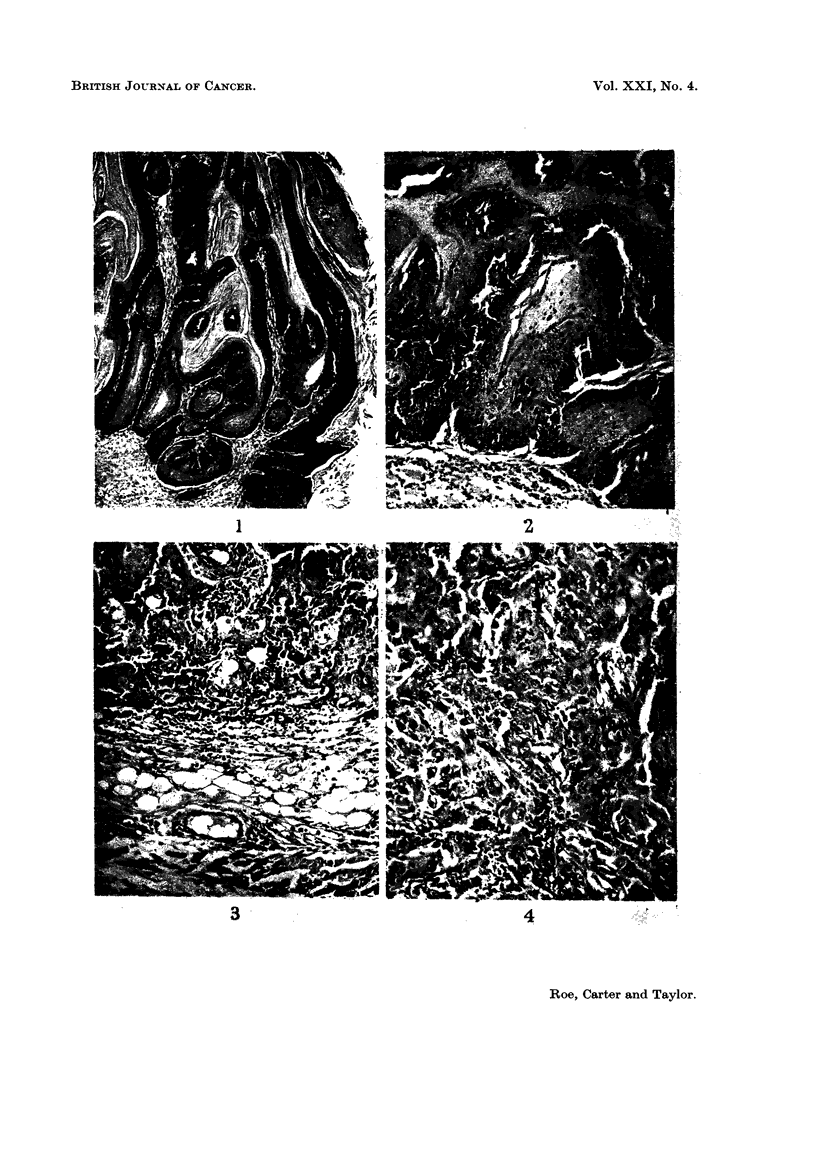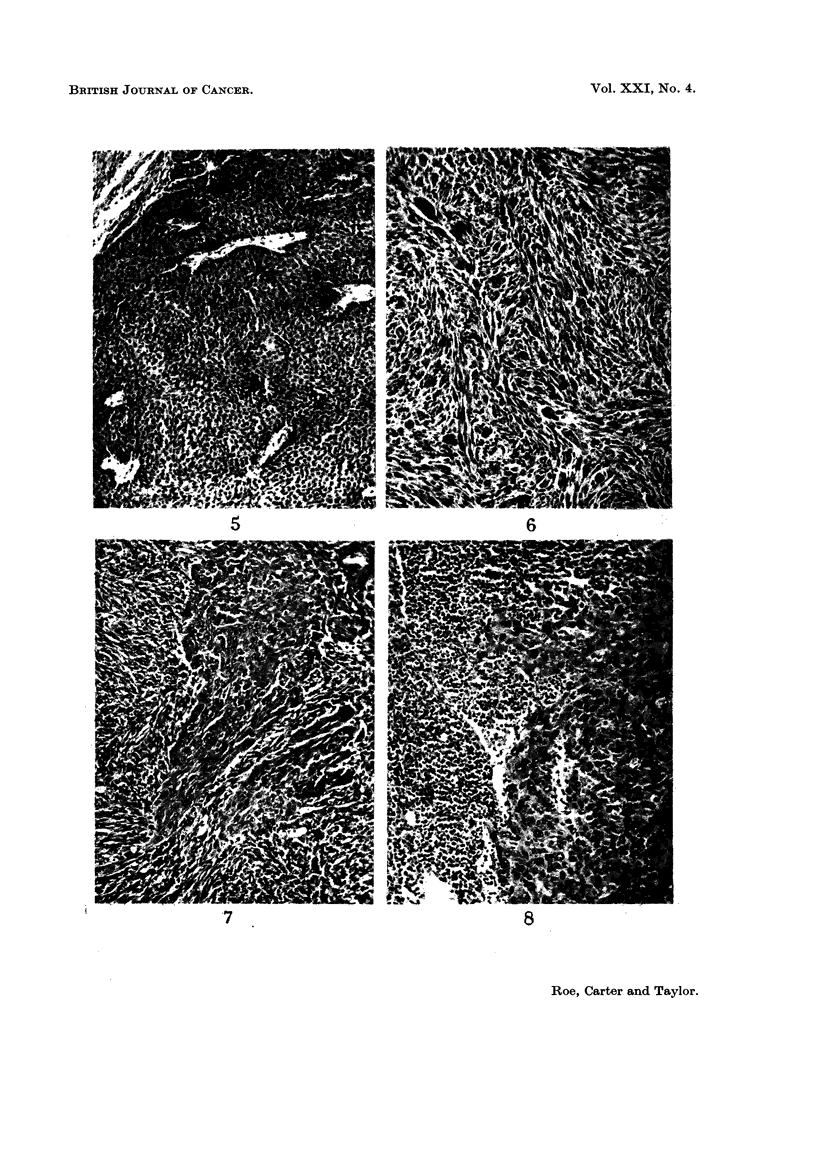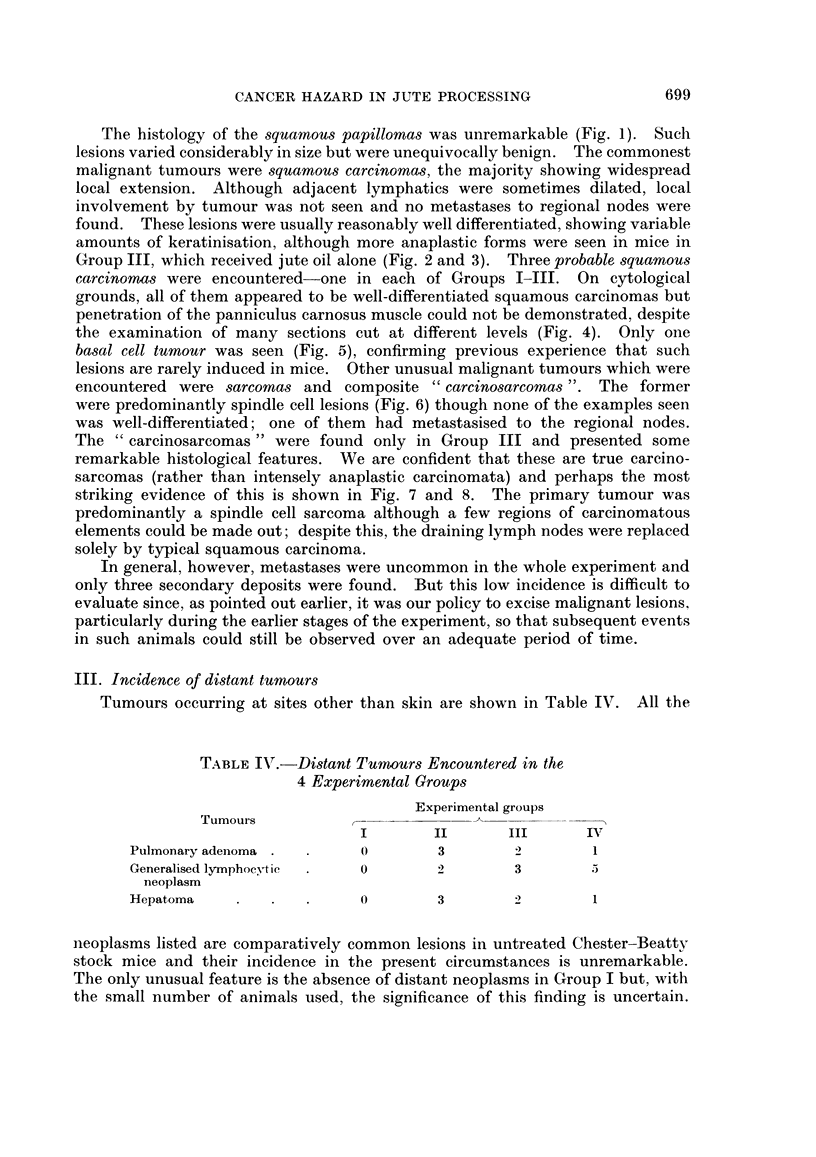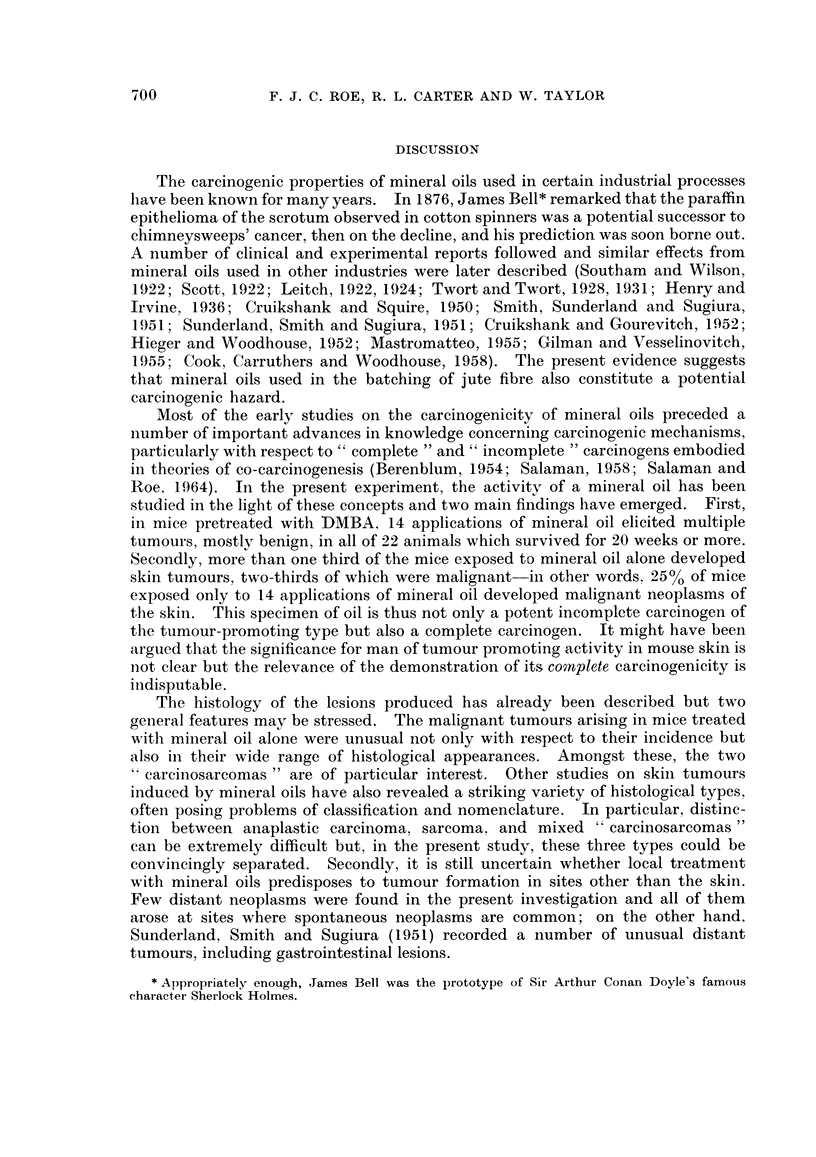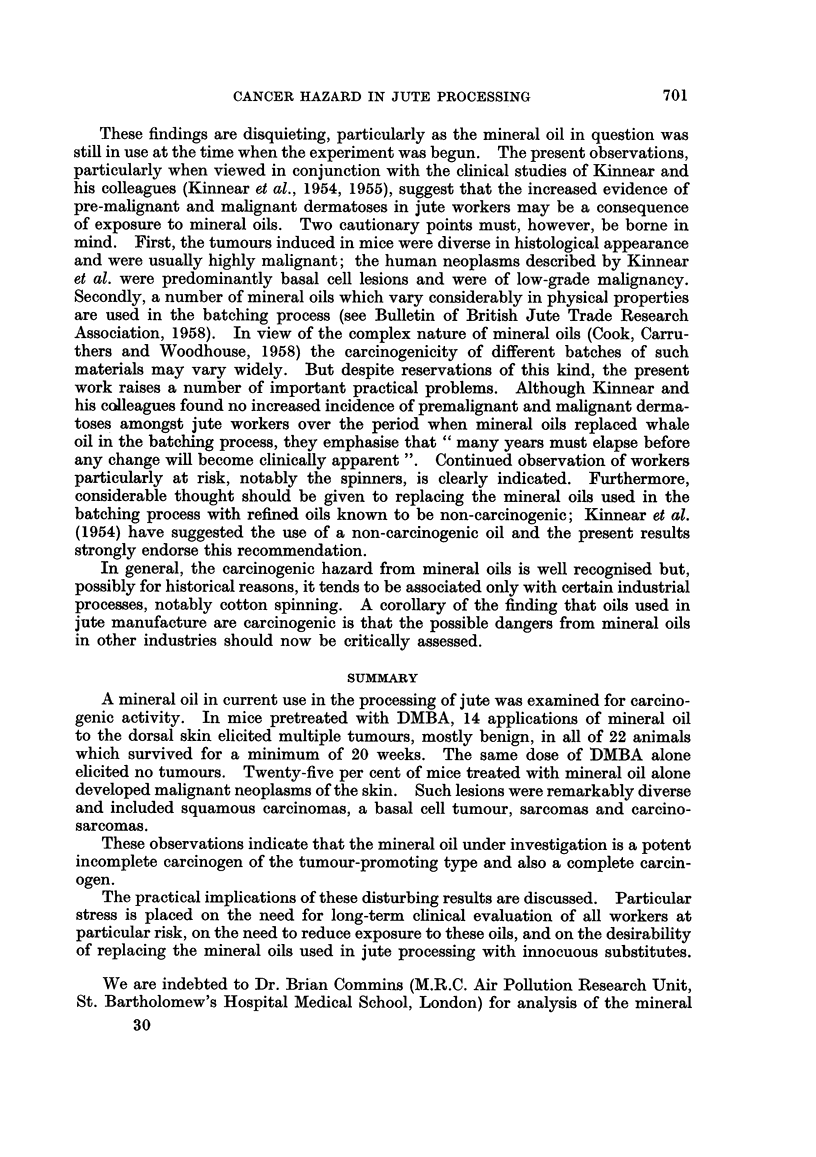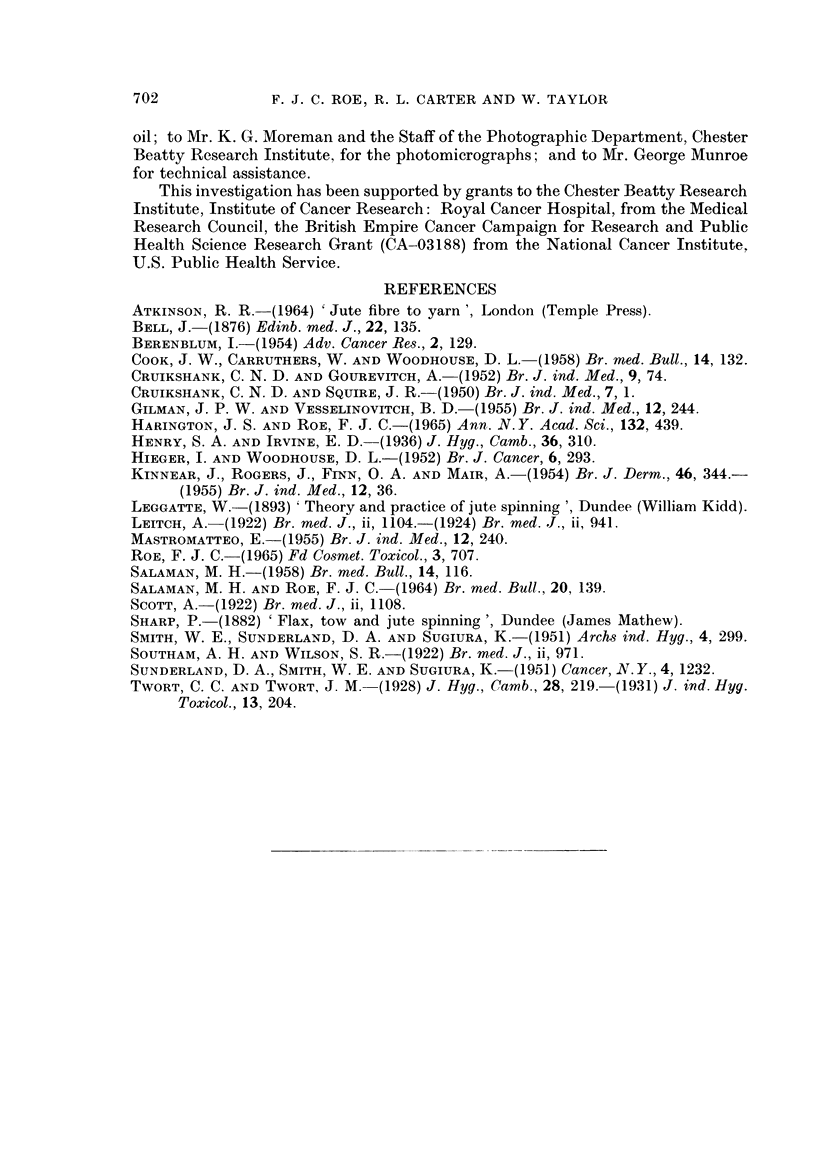# Cancer hazard from mineral oil used in the processing of jute.

**DOI:** 10.1038/bjc.1967.82

**Published:** 1967-12

**Authors:** F. J. Roe, R. L. Carter, W. Taylor

## Abstract

**Images:**


### CANCER HAZARD FROM MINERAL OIL USED IN THE PROCESSING OF JUTE

F. J. C. ROE, R. L. CARTER AND W. TAYLOR

*From the Chester Beatty Research Institute, Institute of Cancer Research: Royal Cancer Hospital, London; and the Department of Public Health and Social Medicine, Queen's College, Dundee*

Received for publication July 6, 1967

JUTE was introduced into Dundee from India between 1830 and 1840. The new fibre was not favourably received by the flax spinners of that time who found it harsh and difficult to handle. All attempts to spin jute alone were unsuccessful and it was found necessary to spin it in combination with flax. Later it was discovered that if jute was treated with an emulsion of whale oil and water, the fibre was softened and lubricated and could then be spun on the existing flax machinery (see Bulletin of British Jute Trade Research Association, 1955). This unusual process was known as " batching "; it is an integral feature of jute manufacture—few other fibres require similar treatment—and it has remained essentially unchanged until the present day although a number of different oils have been used. Thus, in the last century, seal and sperm oils were employed as well as whale oils in Dundee where a whaling fleet was conveniently based on the Tay estuary (see Atkinson, 1964). Later, an emulsion of whale, seal and mineral oils was introduced for the batching of cheaper jute yarns (Sharp, 1882). Whale oils were gradually replaced by the cheaper mineral oils (Legatte, 1893) and, from about 1920 onwards, the quantity of whale oil in use decreased from about 50% to less than 1% in 1966.

Despite the long history of jute processing in the United Kingdom, surprisingly little information is available on the potential health hazards associated with this industry. Kinnear, Rogers, Finn and Mair (1954, 1955) have, however, demonstrated an unusually high incidence of dermatoses amongst jute workers. In particular, pre-malignant degenerative conditions such as " senile " keratosis, pigmentation, purpura and telangiectasia occur with increased frequency and may progress to overt carcinoma. The various lesions are virtually confined to exposed areas of the skin and are most common amongst jute spinners.

The cause of these changes is unknown but in view of the undoubted carcinogenicity of mineral oils used in other processes—notably the manufacture of cotton —the oils used in the batching of jute would seem to be the first agents to examine for carcinogenic activity. Evidence of such activity is now available. In the course of other studies, Harington and Roe (1965) demonstrated that a sample of batching oil was carcinogenic when applied to the dorsal skin of mice and showed that the same material also promoted the development of skin tumours in mice previously exposed to a subcarcinogenic dose of a known carcinogen. These observations prompted a more detailed study of this mineral oil and, in the present paper, we describe its carcinogenic and tumour-promoting effects.

### MATERIAL AND METHODS

*Mice.*—Male Chester-Beatty stock mice were used for the experiment. The animals were random-bred and had been vaccinated against ectromelia at 4 weeks of age. When the mice were 6–10 weeks old, they were distributed in a random fashion into 4 equal groups and thereafter housed 8 per cage on wood shavings in metal boxes. They were fed with cubed Diet No. 86 (obtained from Plowco Feeds Ltd., South Godstone, Surrey) and provided with water *ad libitum.*

*Oil.*—A sample of mineral oil at present in use at a large jute-processing factory in the United Kingdom (Batch No. CBH 24.2.65) was tested. The specifications of the oil were as follows:

| | |
|---|---|
| Specific gravity (at 15·5° C.) | 0·93 |
| Viscosity (Redwood No. 170° F.) | 160–260 seconds |
| Flash point | 330° F. |
| Acid value (mg. KOH/g.) | <0·4 |
| Saponification value | <3·0 |

The oil was examined for aromatic polycyclic hydrocarbons by paper and absorption column chromatography, followed by ultra-violet spectroscopy. 3,4-benzopyrene was present at just detectable levels but the concentration was less than 1 $\mu$g. per g. of oil (*i.e.* <1 p.p.m. on a weight basis). No other polycyclic hydrocarbons were found but it is emphasised that their identification in oils of this kind is extremely difficult.

*Other chemical agents used in the present experiment.*—7,12-dimethylbenz(a)anthracene (DMBA) was obtained from Koch–Light Laboratories, Colnbrook, Bucks; and acetone (analytical reagent grade) from Hopkin and Williams Ltd.

*Application of materials to skin.*—Test substances were applied by calibrated pipettes to an oval area of dorsal skin extending from the interscapular region to the base of the tail. The hair was removed by electric clippers before the first application and thereafter at weekly intervals throughout the experiment.

*Observation of mice.*—The mice were examined individually at weekly intervals and the presence of all skin lesions, inflammatory or neoplastic, which exceeded 1 mm. in diameter was recorded. Tumours regarded as obviously malignant on macroscopic grounds were removed under ether anaesthesia. In this way, it was possible for individual mice to develop more than one malignant tumour sequentially but special care was always taken not to record the recurrence of an incompletely removed tumour as a second primary lesion.

Mice which became sick during the experiment and all those which survived until its termination at 84 weeks were killed with ether. A standard post-mortem examination (Roe, 1965) was made on each animal. All obviously malignant skin tumours, a proportion of benign skin tumours, and all tumours or suspicious lesions seen at other sites were removed and fixed in Bouin's solution. Paraffin sections were cut at 5 $\mu$. and stained with haemotoxylin and eosin and additionally, in some cases, with haematoxylin and Van Gieson, Masson's trichome and with the modified Foot technique for reticulin fibres.

*Group I* constituted a test of the mineral oil for tumour-promoting activity, after a single sub-carcinogenic dose of DMBA had been applied to the skin. It was originally intended to apply undiluted mineral oil twice weekly for a minimum period of 20 weeks, preliminary toxicity tests having indicated that this dose schedule would probably be tolerated. In the event, applications of oil were

### DETAILS OF EXPERIMENT

Details of treatment in the 4 experimental groups are given in Table I.

TABLE I.—*Details of Treatment*

| Group | No. of mice | Primary treatment | Secondary treatment (beginning 3 weeks after completion of primary treatment). |
|---|---|---|---|
| I | 24 | Single application of 150 $\mu$g. DMBA in 0·2 ml. acetone | 14 applications of 0·25 ml. undiluted mineral oil. (Twice weekly for 4 weeks; no treatment for 18 days* then twice weekly for 3 weeks |
| II | 24 | As in Group I | None |
| III | 24 | 0·2 ml. acetone | As in Group I |
| IV | 24 | None | None |

* Treatment suspended because of ulceration of skin.

suspended after 4 weeks because of inflammation and ulceration of the skin. Healing was almost complete after an interval of 18 days and treatment was resumed. Local inflammation reappeared, however, and treatment was finally stopped after 6 further twice-weekly applications. Animals in Group I thus received 14 applications of 0·25 ml. of oil during the course of 9½ weeks.

*Group II* served as a control for Group I, 24 mice being painted with DMBA only.

*Group III* was included as a test of the mineral oil for complete carcinogenicity; preliminary treatment with DMBA was therefore omitted and the mice received acetone only.

*Group IV*, which consisted of untreated control mice, was set up to establish the incidence of spontaneous tumours of skin and of other sites.

### RESULTS

### I. *Development of skin tumours*

The development of skin tumours in the 4 groups of mice is summarised in Table II.

The results in Group I demonstrate that the mineral oil under investigation is a potent tumour-promoting agent in mice pre-treated with DMBA. More than 90% of the animals developed skin tumours and their average time of induction was short. Large numbers of tumours were produced and although benign lesions predominated, malignant neoplasms occurred in half the tumour-bearing animals.

Mice in Group III, treated with mineral oil *alone*, showed a lower incidence of skin neoplasms, particularly of papillomas. Malignant tumours, however, occurred in 6 of the 9 mice which developed skin tumours and some of these lesions were of unusual histological types (*v.i.*). It thus appears that the mineral oil has moderate carcinogenic activity, even in the absence of prior treatment with a tumour-initiating agent.

The results in Group II indicate that DMBA alone, administered in the low (initiating) dose used in the present experiment, had negligible carcinogenic activity. Only 3 skin tumours appeared and one of the two malignant neoplasms

TABLE II.—*Induction oj Skin Tumours by Mineral Oil*

| Experimental Group | Cumulative numbers of mice developing skin tumours per survivors (Weeks after start of treatment) | | | | | | Total skin tumours at 30 weeks | Average induction time of first tumour (weeks) | Number of mice which developed benign or malignant skin tumours before 84 weeks | Number of mice which developed malignant skin tumours before 84 weeks | Total number of malignant and probably malignant tumours (see also Table III) |
|---|---|---|---|---|---|---|---|---|---|---|---|
| | 5 | 10 | 15 | 20 | 25 | 30 | | | | | |
| I | 0/24 | 15/23 | 21/23 | 22/22 | 22/21 | 22/12 | 296 | 11 | 22 | 11* | 14* |
| II | 0/24 | 0/24 | 0/24 | 0/24 | 0/23 | 0/23 | 0 | 44 | 3 | 2 | 2 |
| III | 0/24 | 1/24 | 5/24 | 6/24 | 8/23 | 8/23 | 24 | 20 | 9 | 6 | 6 |
| IV | 0/24 | 0/24 | 0/24 | 0/24 | 0/23 | 0/20 | 0 | 0 | 0 | 0 | 0 |

* These totals include two mice in Group I each of which developed an obvious carcinoma, diagnosed macroscopically. Both animals died unexpectedly and the tumours were too autolysed for histological examination.

arose on the cheek, at some distance from the treated area. Furthermore, the average induction time was notably longer than that for Groups I or III.

No skin tumours were seen among the control mice in Group IV.

### II. *Histology of skin tumours*

Skin tumours selected for microscopical examination because of suspected malignancy or for other reasons (see Materials and Methods) were classified within the following groups: BENIGN TUMOURS (squamous papilloma); PROBABLY MALIGNANT TUMOURS; and MALIGNANT TUMOURS (squamous carcinoma, basal cell tumour, "carcinosarcoma", sarcoma). Three of these terms need to be clarified. (1) All tumours classified as malignant had invaded the panniculus carnosus muscle. (2) The category of probably malignant tumour was reserved for a small number of neoplasms which showed cellular atypia, absence of delimiting basement-membrane and invasion of dermal structures but which had *not* yet reached the panniculus carnosus muscle: (3) " Carcinosarcoma " was used in a purely descriptive fashion to characterise tumours which were apparently composed of both carcinomatous and sarcomatous elements.

The distribution of these various types of neoplasm within the experimental groups is shown in Table III while their histological features are illustrated in Fig. 1 to 8.

TABLE III.—*Distribution of Tumours Examined Histologically*

| Skin tumours | Experimental groups: Group I DMBA+ mineral oil | Group II DMBA alone | Group III mineral oil alone | Group IV untreated controls |
|---|---|---|---|---|
| Total number of tumours examined | 34 | 3 | 8 | 0 |
| BENIGN | | | | |
| Squamous papilloma | 22 | 1 | 2 | 0 |
| PROBABLY MALIGNANT | | | | |
| Squamous tumour | 1 | 1 | 1 | 0 |
| MALIGNANT | | | | |
| Squamous carcinoma | 9 | 1 | 2 | 0 |
| Basal cell tumour | 1 | 0 | 0 | 0 |
| " Carcinosarcoma " | 0 | 0 | 2 | 0 |
| Sarcoma | 1 | 0 | 1 | 0 |

EXPLANATION OF PLATES.

All photomicrographs are of sections stained with haematoxylin and eosin at × 115 unless otherwise stated.

FIG. 1.—Mouse, Group I. Squamous papilloma; a keratinising lesion showing no evidence of malignancy. × 40.

FIG. 2.—Mouse, Group I. Squamous carcinoma; a well-differentiated keratinising lesion which is beginning to erode the panniculus carnosus.

FIG. 3.—Mouse, Group III. Probable squamous carcinoma; this tumour is not well differentiated but it has not yet infiltrated the panniculus carnosus.

FIG. 4.—Mouse, Group III. Rather poorly differentiated squamous carcinoma which has widely infiltrated the dermis.

FIG. 5.—Mouse, Group III. Basal cell tumour.

FIG. 6.—Mouse, Group III. Sarcoma, composed mainly of spindle cells

FIG. 7.—Mouse, Group III. " Carcinosarcoma ". The tumour is composed mainly of sarcomatous elements but also contains " islands " of carcinoma.

FIG. 8.—Mouse, Group III. Regional lymph nodes draining the tumour shown in Fig. 7. The node is largely replaced by squamous carcinoma; no sarcomatous elements seen.

1

2

3

4

5

6

7

8

The histology of the *squamous papillomas* was unremarkable (Fig. 1). Such lesions varied considerably in size but were unequivocally benign. The commonest malignant tumours were *squamous carcinomas*, the majority showing widespread local extension. Although adjacent lymphatics were sometimes dilated, local involvement by tumour was not seen and no metastases to regional nodes were found. These lesions were usually reasonably well differentiated, showing variable amounts of keratinisation, although more anaplastic forms were seen in mice in Group III, which received jute oil alone (Fig. 2 and 3). Three *probable squamous carcinomas* were encountered—one in each of Groups I–III. On cytological grounds, all of them appeared to be well-differentiated squamous carcinomas but penetration of the panniculus carnosus muscle could not be demonstrated, despite the examination of many sections cut at different levels (Fig. 4). Only one *basal cell tumour* was seen (Fig. 5), confirming previous experience that such lesions are rarely induced in mice. Other unusual malignant tumours which were encountered were *sarcomas* and composite " *carcinosarcomas* ". The former were predominantly spindle cell lesions (Fig. 6) though none of the examples seen was well-differentiated; one of them had metastasised to the regional nodes. The " carcinosarcomas " were found only in Group III and presented some remarkable histological features. We are confident that these are true carcinosarcomas (rather than intensely anaplastic carcinomata) and perhaps the most striking evidence of this is shown in Fig. 7 and 8. The primary tumour was predominantly a spindle cell sarcoma although a few regions of carcinomatous elements could be made out; despite this, the draining lymph nodes were replaced solely by typical squamous carcinoma.

In general, however, metastases were uncommon in the whole experiment and only three secondary deposits were found. But this low incidence is difficult to evaluate since, as pointed out earlier, it was our policy to excise malignant lesions, particularly during the earlier stages of the experiment, so that subsequent events in such animals could still be observed over an adequate period of time.

### III. *Incidence of distant tumours*

Tumours occurring at sites other than skin are shown in Table IV. All the

TABLE IV.—*Distant Tumours Encountered in the 4 Experimental Groups*

| Tumours | Experimental groups | | | |
|---|---|---|---|---|
| | I | II | III | IV |
| Pulmonary adenoma . . | 0 | 3 | 2 | 1 |
| Generalised lymphocytic neoplasm . | 0 | 2 | 3 | 5 |
| Hepatoma . . . . | 0 | 3 | 2 | 1 |

neoplasms listed are comparatively common lesions in untreated Chester–Beatty stock mice and their incidence in the present circumstances is unremarkable. The only unusual feature is the absence of distant neoplasms in Group I but, with the small number of animals used, the significance of this finding is uncertain.

### DISCUSSION

The carcinogenic properties of mineral oils used in certain industrial processes have been known for many years. In 1876, James Bell* remarked that the paraffin epithelioma of the scrotum observed in cotton spinners was a potential successor to chimneysweeps' cancer, then on the decline, and his prediction was soon borne out. A number of clinical and experimental reports followed and similar effects from mineral oils used in other industries were later described (Southam and Wilson, 1922; Scott, 1922; Leitch, 1922, 1924; Twort and Twort, 1928, 1931; Henry and Irvine, 1936; Cruikshank and Squire, 1950; Smith, Sunderland and Sugiura, 1951; Sunderland, Smith and Sugiura, 1951; Cruikshank and Gourevitch, 1952; Hieger and Woodhouse, 1952; Mastromatteo, 1955; Gilman and Vesselinovitch, 1955; Cook, Carruthers and Woodhouse, 1958). The present evidence suggests that mineral oils used in the batching of jute fibre also constitute a potential carcinogenic hazard.

Most of the early studies on the carcinogenicity of mineral oils preceded a number of important advances in knowledge concerning carcinogenic mechanisms, particularly with respect to " complete " and " incomplete " carcinogens embodied in theories of co-carcinogenesis (Berenblum, 1954; Salaman, 1958; Salaman and Roe, 1964). In the present experiment, the activity of a mineral oil has been studied in the light of these concepts and two main findings have emerged. First, in mice pretreated with DMBA, 14 applications of mineral oil elicited multiple tumours, mostly benign, in all of 22 animals which survived for 20 weeks or more. Secondly, more than one third of the mice exposed to mineral oil alone developed skin tumours, two-thirds of which were malignant—in other words, 25% of mice exposed only to 14 applications of mineral oil developed malignant neoplasms of the skin. This specimen of oil is thus not only a potent incomplete carcinogen of the tumour-promoting type but also a complete carcinogen. It might have been argued that the significance for man of tumour promoting activity in mouse skin is not clear but the relevance of the demonstration of its *complete* carcinogenicity is indisputable.

The histology of the lesions produced has already been described but two general features may be stressed. The malignant tumours arising in mice treated with mineral oil alone were unusual not only with respect to their incidence but also in their wide range of histological appearances. Amongst these, the two " carcinosarcomas " are of particular interest. Other studies on skin tumours induced by mineral oils have also revealed a striking variety of histological types, often posing problems of classification and nomenclature. In particular, distinction between anaplastic carcinoma, sarcoma, and mixed " carcinosarcomas " can be extremely difficult but, in the present study, these three types could be convincingly separated. Secondly, it is still uncertain whether local treatment with mineral oils predisposes to tumour formation in sites other than the skin. Few distant neoplasms were found in the present investigation and all of them arose at sites where spontaneous neoplasms are common; on the other hand, Sunderland, Smith and Sugiura (1951) recorded a number of unusual distant tumours, including gastrointestinal lesions.

* Appropriately enough, James Bell was the prototype of Sir Arthur Conan Doyle's famous character Sherlock Holmes.

These findings are disquieting, particularly as the mineral oil in question was still in use at the time when the experiment was begun. The present observations, particularly when viewed in conjunction with the clinical studies of Kinnear and his colleagues (Kinnear *et al.*, 1954, 1955), suggest that the increased evidence of pre-malignant and malignant dermatoses in jute workers may be a consequence of exposure to mineral oils. Two cautionary points must, however, be borne in mind. First, the tumours induced in mice were diverse in histological appearance and were usually highly malignant; the human neoplasms described by Kinnear *et al.* were predominantly basal cell lesions and were of low-grade malignancy. Secondly, a number of mineral oils which vary considerably in physical properties are used in the batching process (see Bulletin of British Jute Trade Research Association, 1958). In view of the complex nature of mineral oils (Cook, Carruthers and Woodhouse, 1958) the carcinogenicity of different batches of such materials may vary widely. But despite reservations of this kind, the present work raises a number of important practical problems. Although Kinnear and his colleagues found no increased incidence of premalignant and malignant dermatoses amongst jute workers over the period when mineral oils replaced whale oil in the batching process, they emphasise that " many years must elapse before any change will become clinically apparent ". Continued observation of workers particularly at risk, notably the spinners, is clearly indicated. Furthermore, considerable thought should be given to replacing the mineral oils used in the batching process with refined oils known to be non-carcinogenic; Kinnear *et al.* (1954) have suggested the use of a non-carcinogenic oil and the present results strongly endorse this recommendation.

In general, the carcinogenic hazard from mineral oils is well recognised but, possibly for historical reasons, it tends to be associated only with certain industrial processes, notably cotton spinning. A corollary of the finding that oils used in jute manufacture are carcinogenic is that the possible dangers from mineral oils in other industries should now be critically assessed.

### SUMMARY

A mineral oil in current use in the processing of jute was examined for carcinogenic activity. In mice pretreated with DMBA, 14 applications of mineral oil to the dorsal skin elicited multiple tumours, mostly benign, in all of 22 animals which survived for a minimum of 20 weeks. The same dose of DMBA alone elicited no tumours. Twenty-five per cent of mice treated with mineral oil alone developed malignant neoplasms of the skin. Such lesions were remarkably diverse and included squamous carcinomas, a basal cell tumour, sarcomas and carcinosarcomas.

These observations indicate that the mineral oil under investigation is a potent incomplete carcinogen of the tumour-promoting type and also a complete carcinogen.

The practical implications of these disturbing results are discussed. Particular stress is placed on the need for long-term clinical evaluation of all workers at particular risk, on the need to reduce exposure to these oils, and on the desirability of replacing the mineral oils used in jute processing with innocuous substitutes.

We are indebted to Dr. Brian Commins (M.R.C. Air Pollution Research Unit, St. Bartholomew's Hospital Medical School, London) for analysis of the mineral oil; to Mr. K. G. Moreman and the Staff of the Photographic Department, Chester Beatty Research Institute, for the photomicrographs; and to Mr. George Munroe for technical assistance.

This investigation has been supported by grants to the Chester Beatty Research Institute, Institute of Cancer Research: Royal Cancer Hospital, from the Medical Research Council, the British Empire Cancer Campaign for Research and Public Health Science Research Grant (CA–03188) from the National Cancer Institute, U.S. Public Health Service.

### REFERENCES

ATKINSON, R. R.—(1964) 'Jute fibre to yarn', London (Temple Press).
BELL, J.—(1876) *Edinb. med. J.*, **22**, 135.
BERENBLUM, I.—(1954) *Adv. Cancer Res.*, **2**, 129.
COOK, J. W., CARRUTHERS, W. AND WOODHOUSE, D. L.—(1958) *Br. med. Bull.*, **14**, 132.
CRUIKSHANK, C. N. D. AND GOUREVITCH, A.—(1952) *Br. J. ind. Med.*, **9**, 74.
CRUIKSHANK, C. N. D. AND SQUIRE, J. R.—(1950) *Br. J. ind. Med.*, **7**, 1.
GILMAN, J. P. W. AND VESSELINOVITCH, B. D.—(1955) *Br. J. ind. Med.*, **12**, 244.
HARINGTON, J. S. AND ROE, F. J. C.—(1965) *Ann. N.Y. Acad. Sci.*, **132**, 439.
HENRY, S. A. AND IRVINE, E. D.—(1936) *J. Hyg., Camb.*, **36**, 310.
HIEGER, I. AND WOODHOUSE, D. L.—(1952) *Br. J. Cancer*, **6**, 293.
KINNEAR, J., ROGERS, J., FINN, O. A. AND MAIR, A.—(1954) *Br. J. Derm.*, **46**, 344.—(1955) *Br. J. ind. Med.*, **12**, 36.
LEGGATTE, W.—(1893) 'Theory and practice of jute spinning', Dundee (William Kidd).
LEITCH, A.—(1922) *Br. med. J.*, ii, 1104.—(1924) *Br. med. J.*, ii, 941.
MASTROMATTEO, E.—(1955) *Br. J. ind. Med.*, **12**, 240.
ROE, F. J. C.—(1965) *Fd Cosmet. Toxicol.*, **3**, 707.
SALAMAN, M. H.—(1958) *Br. med. Bull.*, **14**, 116.
SALAMAN, M. H. AND ROE, F. J. C.—(1964) *Br. med. Bull.*, **20**, 139.
SCOTT, A.—(1922) *Br. med. J.*, ii, 1108.
SHARP, P.—(1882) 'Flax, tow and jute spinning', Dundee (James Mathew).
SMITH, W. E., SUNDERLAND, D. A. AND SUGIURA, K.—(1951) *Archs ind. Hyg.*, **4**, 299.
SOUTHAM, A. H. AND WILSON, S. R.—(1922) *Br. med. J.*, ii, 971.
SUNDERLAND, D. A., SMITH, W. E. AND SUGIURA, K.—(1951) *Cancer, N.Y.*, **4**, 1232.
TWORT, C. C. AND TWORT, J. M.—(1928) *J. Hyg., Camb.*, **28**, 219.—(1931) *J. ind. Hyg. Toxicol.*, **13**, 204.